# Calciprotein particles in cats with naturally occurring chronic kidney disease

**DOI:** 10.1093/jvimsj/aalag037

**Published:** 2026-03-10

**Authors:** Pak-Kan Tang, Makoto Kuro-o, Miki Tsuchida, Rebecca F Geddes, Rosanne E Jepson, Yu-Mei Chang, Jonathan Elliott

**Affiliations:** Department of Comparative Biomedical Sciences, Royal Veterinary College, University of London, London, United Kingdom; Division of Mineral Metabolism, Center for Molecular Medicine, Jichi Medical University, Shimotsuke, Tochigi, Japan; Division of Mineral Metabolism, Center for Molecular Medicine, Jichi Medical University, Shimotsuke, Tochigi, Japan; Department of Clinical Science and Services, Royal Veterinary College, University of London, London, United Kingdom; Department of Clinical Science and Services, Royal Veterinary College, University of London, London, United Kingdom; Department of Comparative Biomedical Sciences, Royal Veterinary College, University of London, London, United Kingdom; Department of Comparative Biomedical Sciences, Royal Veterinary College, University of London, London, United Kingdom

**Keywords:** calcification propensity, calciprotein particle, CKD-MBD, fibroblast growth factor-23, mineralization, phosphate

## Abstract

**Background:**

Calciprotein particles (CPP) are nanoparticles that play an important role in the pathogenesis of chronic kidney disease-mineral and bone disorder (CKD-MBD).

**Hypothesis/Objectives:**

Identification of plasma CPP and preliminary exploration of the relationships among CPP concentrations, calcification propensity (T_50_), and CKD-MBD variables in cats with azotemic CKD.

**Animals:**

Cats with azotemic CKD (*n* = 52) stabilized on a phosphate-restricted diet (PRD).

**Methods:**

Total CPP (T-CPP), low-density CPP (L-CPP), and high-density CPP (H-CPP) were measured in heparinized plasma using a fluorescent bisphosphonate (OsteoSense) after gel filtration. Standardized linear regression models evaluated associations among CPP, T_50_, and CKD-MBD variables. Generalized estimating equations compared preprandial and postprandial CPP concentrations. Calciprotein particle changes (ΔCPP) between visits were compared between cats with different ionized calcium (iCa) trajectories using independent samples *t-*test or Mann–Whitney *U* tests.

**Results:**

Fibroblast growth factor-23 (standardized coefficient [sβ], 0.35; *P* = .04) and parathyroid hormones (sβ, −0.34; *P* = .042) were significantly associated with preprandial T-CPP concentrations in cats fed a PRD, whereas phosphate was significantly associated with postprandial T-CPP (sβ, 0.72; *P* = .003) and L-CPP (sβ, 0.75; *P* = .003) concentrations before dietary phosphate restriction. ΔT-CPP was significantly greater in cats with CKD with uptrend iCa compared to those with downtrend iCa after PRD stabilization (14 105 ± 36 299 AU vs −29 495 ± 49 664 AU; *P* = .036).

**Conclusions and clinical importance:**

Calciprotein particle measurement is possible in cats and adds to the assessment of CKD-MBD, particularly the risk of soft tissue mineralization. The trajectory of iCa after PRD might influence CPP concentrations in cats with CKD.

## Introduction

**Figure 1:**
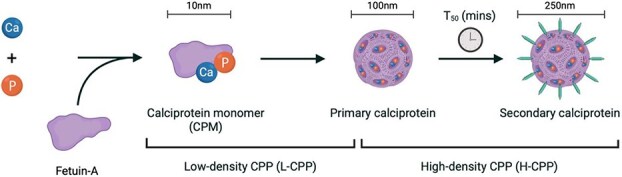
Formation and transformation process of CPP. Abbreviation: CPP = calciprotein particles. *Figure created with*  BioRender.com.

Fetuin-A is a circulating multifunctional glycoprotein produced by hepatocytes, which plays a key role in the regulation of biomineralization as a potent inhibitor of vascular calcification (VC).^1,2^ Aggregation of fetuin-A with calcium-phosphate mineral allows calciprotein monomers (CPM) to form. Calciprotein monomers are small nanoparticles circulating in colloidal suspension with a size up to 10 nm in diameter.^3,4^ Calciprotein monomers integrate with additional proteins to consolidate into amorphous spherical aggregates with diameter ranging from 50 to 100 nm; designated as primary calciprotein particles (CPP-1).^5–7^ Primary CPP might undergo structural and compositional rearrangement and transform spontaneously into pro-inflammatory crystalline secondary CPP (CPP-2) over time.^4,8^ Secondary CPP are elongated, ellipsoid, and larger in size, approximately 100-250 nm in long-axis diameter, with a compact crystalline core surrounded by a protein layer that displays a needle-shaped structure.^8–10^ A schematic diagram illustrating the formation and transformation process of CPP is presented in Figure 1. This resulting morphological change is reminiscent of hydroxyapatite; it allows the crystalline CPP-2 to be more thermodynamically stable and capable of inducing inflammation and calcification in cultured vascular smooth muscle cells.^11,12^ In addition to classifying CPP based on their crystallinity, another way to classify these particles is based on their density.^13^ Low-density CPP (L-CPP) are considered to consist of CPM and small CPP-1, whereas high-density CPP (H-CPP) encompass large CPP-1 and CPP-2.^14^

Both CPM and CPP-1 are considered to be harmless and even protective by acting as a barrier to limit precipitation of insoluble calcium-phosphate crystals in the blood, and these particles are easily cleared from the circulation under normal physiological conditions. Calciprotein monomers are predominantly cleared by the kidneys through glomerular filtration, and either dissociate in the urine to fetuin-A and calcium phosphate mineral or are endocytosed and dissociated in the lysosome,^4^ while CPP-1 are cleared by liver sinusoidal endothelial cells.^9^ Primary CPP are suggested to promote fibroblast growth factor-23 (FGF-23) production in osteoblasts.^15^ Nevertheless, in conditions that disturb mineral homeostasis and result in hypercalcemia or hyperphosphatemia, or when the clearance process of CPP is impaired such as with chronic kidney disease (CKD), accumulation of CPP, especially the mature crystalline CPP-2 through the amorphous-to-crystalline transition via Ostwald ripening, might occur.^7,16^ This is commonly observed in humans with CKD.^13,17–19^

In humans with end-stage renal disease, hyperphosphatemia is an established risk factor for VC and poor prognosis.^20–22^ Similar findings are reported in cats and dogs with azotemic CKD.^23–25^ In addition to serum phosphate, serum calcium, and calcium-phosphate product (CaPP) is associated with greater risk of VC and all-cause mortality in humans with CKD.^26–29^ Therefore, high concentrations of circulating CPP, consisting predominantly of calcium-phosphate minerals, might play an important pathophysiological role in the complications and death in humans with CKD.

Calciprotein particles have attracted increasing research interest in the field of human nephrology over the past decade. Similar to humans, cats develop spontaneous CKD, and MBD is a major complicating factor associated with progression and death in cats with CKD. Hence, the aims of our study were to (i) explore the use of a novel assay for detecting and quantifying plasma CPP concentrations in cats with naturally occurring CKD stabilized on a standardized phosphate-restricted diet (PRD); (ii) evaluate the relationships between CPP concentrations and CKD-MBD variables; and (iii) compare preprandial CPP concentrations between cats with different trajectories of blood ionized calcium (iCa) concentration after dietary phosphate restriction.

## Materials and methods

### Study cohort

Cats included in this retrospective study were originally enrolled in an ongoing longitudinal observational study for which informed consent of the owner was obtained and approval of the Ethics and Welfare Committee of the Royal Veterinary College (RVC) was granted (URN 2023 2225-3). For this present study, clinical records of the RVC Ageing Cat Clinic, which was held in 2 first-opinion practices in London, between January 1st, 2010 and May 31st, 2022 were reviewed and cats with a diagnosis of azotemic CKD with specific sample criteria were identified.

Under the protocol of the longitudinal observational study, cats were diagnosed with azotemic CKD on the basis of plasma creatinine concentration ≥ 2 mg/dL in conjunction with inappropriately diluted urine (urine specific gravity < 1.035), or plasma creatinine concentration ≥ 2 mg/dL on 2 consecutive occasions 2-4 weeks apart without evidence of prerenal cause. Azotemic cats with CKD were routinely offered a PRD and re-evaluated with blood and where possible urine samples obtained after approximately 28-42 days. Subsequent monitoring was offered approximately every 56 days, including physical examination and blood pressure assessment, with blood and urine sampling (where possible) performed at every other visit.

Blood samples were collected by jugular venipuncture into heparinized, ethylenediaminetetraacetic acid (EDTA) and plain tubes, whereas urine was obtained by cystocentesis. Measurements of iCa concentration were obtained immediately using non-anticoagulated whole blood, or using electrolyte-balanced heparinized whole blood within 5 min of venipuncture,^30^ using a point-of-care blood analyzer (*i-STAT 1*, Abbott Point of Care, Inc, Princeton, New Jersey). All samples collected were stored at 4°C for < 6 h before centrifugation and separation. Biochemical analyses were performed on heparinized plasma by an external laboratory (IDEXX laboratories, Wetherby, UK). Residual blood and urine samples were initially stored at –20°C before being transferred to –80°C for future batch analysis. Stored EDTA plasma samples were used to measure intact FGF-23 using a validated ELISA (FGF-23 ELISA Kit, Kainos Laboratories, Tokyo, Japan)^31^ and parathyroid hormone (PTH) was measured using either a validated total intact PTH immunoradiometric assay^32^ (IRA; total intact PTH immunoradiometric assay-coated bead version, 3KG600, Scantibodies, Santee, California) or a validated 2-site immunoenzymatic assay^33^ (IEA; ST AIA-PACK Intact PTH, Tosoh Bioscience, Tessenderlo, Belgium). The PTH measurements obtained using the 2 assays were comparable (Figure S1). Parathyroid hormone concentrations of 2.6 and 0.55 pg/mL were assigned to samples measured at < 5.2 and < 1.1 pg/mL, the lower limit of detection of the IRA and IEA, respectively.^32,33^

#### Selection of cats

A previously unthawed frozen heparinized plasma sample had to be available for the measurement of CPP from azotemic cats with CKD that had been stabilized and monitored after transition to PRD (Feline Veterinary Diet Renal [dry and wet], Royal Canin SAS, Aimargues, France [dry], and Masterfoods, Bruck, Austria [wet]), with a phosphorus content of 0.7-1.1 g/Mcal and calcium-to-phosphorus ratio (Ca:P) of 1.3-1.9. For this study, dietary stabilization was defined as eating ≥ 50% in proportion by volume of the total food intake since the initiation of a PRD for a minimum of 4 weeks.

#### Case selection for evaluation of variables associated with CPP

Cats were included in evaluation of variables associated with CPP if a suitable sample was available for CPP evaluation, the cat had been successfully stabilized on PRD and clinical records indicated that food had been withheld from the cat for a minimum period of 8 h before collection of the available sample.

#### Case selection for evaluation of effect of iCa status on CPP

Cats were included in the evaluation of effect of iCa status if there was > 1 follow-up visit where iCa status was available after transition and successful stabilization on PRD. The visits were classified as follows: first follow-up visit after the initiation of a PRD was designated as “follow-up visit A” and the subsequent as “follow-up visit B.” Both samples were required to have been obtained after food had been withheld for a minimum of 8 h. Cats with a higher iCa at follow-up visit B compared to follow-up visit A were classified as having an uptrend iCa status, whereas those with a lower iCa at follow-up visit B were classified as having a downtrend iCa status.

#### Case inclusion for comparison of CPP in preprandial and postprandial state

Identified azotemic cats with CKD were reviewed to ascertain availability of previously unthawed heparinized plasma samples, collected before introduction of a PRD. Samples were selected from cats where either the owner could confirm food had been withheld for a minimum of 8 h or where blood samples had been collected postprandially due to owner omission to withhold food. In a small subset of cats, 2 pre-PRD samples were available, allowing paired comparison between preprandial and postprandial states.

#### Exclusion criteria

Cats were excluded if hyperthyroidism was suspected clinically and their plasma total thyroxine concentration was > 40 nmol/L, if they were undergoing medical therapy for hyperthyroidism, had diabetes mellitus, or were being treated with corticosteroids, bisphosphonates, or phosphate binders. Cats with systemic hypertension managed with a stable dose of amlodipine besylate were eligible for inclusion.

Since this study was considered as an exploratory study and sample size was determined by the availability of stored samples that met the inclusion and exclusion criteria, a priori power or sample size calculation was not performed.

### Data collection

#### Signalments and CKD-MBD variables

Clinical records from the cats with CKD were reviewed, with the following historical and biochemical data extracted: age, sex, plasma concentrations of total calcium (tCa), phosphate, creatinine, symmetric dimethylarginine (SDMA), urea, total magnesium (tMg), total protein, albumin, FGF-23, PTH, blood iCa concentration, and PCV.

#### Calciprotein particles (CPP) and T_50_ measurement

Identification and quantification of CPP was performed on stored heparinized plasma by M.K. and M.T. at Jichi Medical University, Japan using the gel-filtration method as previously described.^13^ In brief, frozen samples stored at –80°C were first thawed and incubated at 25°C for 24 h to stimulate the ripening process to transform amorphous CPP-1 to crystalline CPP-2, then stored frozen again at –80°C for future analysis. Before starting the CPP assay, samples were thawed and divided into 2 tubes, one of which was centrifuged at 16 000 *g* for 2 h at 25°C to separate L-CPP (supernatant) from H-CPP (sediment) while the other tube was incubated at 25°C statically (for T-CPP). An infrared fluorescent bisphosphonate (OsteoSense 680EX, PerkinElmer Inc., Waltham, MA, USA), that binds to crystalline calcium phosphate, was added to plasma samples and incubated at 25°C for 60 min. The sample was subsequently applied to a gel filtration spin column with a molecular cut-off at 40 kDa and centrifuged at 1000 *g* for 2 min to remove unbound OsteoSense. The amount of T-CPP and L-CPP in the samples was determined by the fluorescence intensity of OsteoSense using an infrared fluorescence scanner. The H-CPP concentration was calculated by subtracting the concentration of L-CPP from the T-CPP. When the calculated H-CPP was < 0 arbitrary unit (AU), H-CPP was designated as 0 AU in that sample. Calcification propensity was determined by plasma T_50_ values which was quantified using the published method.^34^ Phosphate and calcium were added to the plasma samples at final concentrations of 6 and 10 mM, respectively, to induce supersaturation, promoting the formation of CPP-1, and spontaneous transformation of CPP-1 to CPP-2. The transition time from CPP-1 to CPP-2 was defined as the time between the addition of calcium/phosphate and the observed turbidity increase during a thermo-constant incubation at 37°C. Turbidity measurement was performed by an automated laser-based microplate nephelometer (NEPHELOstar; BMG Labteck, Offenberg, Germany). T_50_ was defined as the time required for each plasma sample to reach half-maximal transformation from CPP-1 to CPP-2.

### Statistical analyses

Statistical analyses were performed using R software (R 4.1.1 GUI 1.77 High Sierra build, R Foundation for Statistical Computing, Vienna, Austria). Type I error rate was set at 0.05.

#### Evaluation of variables associated with preprandial concentrations of CPP and T_50_

Univariable and multivariable linear regression analyses were performed to identify independent variables for T-CPP, L-CPP and detectable H-CPP, and T_50_, with all variables standardized using Z-score transformation before modeling. Age, tCa, iCa, creatinine, urea, SDMA, phosphate, CaPP, tMg, total protein, albumin, log-transformed FGF-23 (ln[FGF-23]), log-transformed PTH (ln[PTH]), and PCV were entered as continuous variables for univariable analyses. Variables associated with the concentrations of different forms of CPP and T_50_ with *P* ≤ .1 were entered into a multivariable model. To mitigate collinearity, tCa was excluded when iCa was included, CaPP was excluded when either tCa or phosphate were included, and urea and SDMA were excluded when creatinine was included. Final models were derived by manual backward elimination, retaining variables with a *P*-value ≤ .1. Results are shown as standardized regression coefficients (sβ, 95% CI). This approach allows comparison between variables, as the standardized coefficients represent the change in the explanatory variable, expressed in SDs, for each standard deviation increase in the outcome variable. The SD and calculation steps used to back-transform sβ to regression coefficients (β) on the original measurement scale are outlined in the Supplementary Material.

Since the majority of cats had nondetectable H-CPP concentrations, a 2-part model was implemented to analyze the variables associated with the presence of detectable H-CPP and its subsequent concentration separately. First a binomial logistic regression model was used to evaluate variables associated with detectable H-CPP. Subsequently, a standardized linear regression model (as detailed above) was performed to identify variables associated with the concentrations of H-CPP in the subset of cats with detectable H-CPP.

A subanalysis was performed to identify variables associated with ln[FGF-23]. Age, tCa, iCa, creatinine, urea, SDMA, phosphate, CaPP, tMg, total protein, albumin, ln[PTH], PCV, and only 1 of the CPP forms (either T-CPP, L-CPP, or H-CPP due to collinearity issue) were entered as continuous variables for multivariable analyses.

#### Comparison of preprandial CPP concentrations between cats with different trajectories of iCa after dietary phosphate restriction

Different forms of CPP at follow-up visit A and follow-up visit B, and the change in CPP (ΔCPP) between visits were compared between iCa groups (downtrend iCa vs uptrend iCa) using either the independent samples *t-*test or Mann–Whitney *U* tests. For each iCa group, ΔCPP was compared by either the paired *t*-test or Wilcoxon signed-ranked test.

#### Comparisons of preprandial and postprandial CPP concentrations in cats with CKD before dietary phosphate restriction

Different forms of CPP and associated CKD-MBD variables measured under different conditions (preprandial vs postprandial) before the initiation of a PRD were compared using generalized estimating equations with exchangeable working correlation matrix to account for cats with paired samples while also incorporated cats with single observations in the dataset. Calciprotein particle concentrations were log-transformed due to the non-normal distribution, and the normality of model residuals was assessed using Q-Q plots.

#### Correlations of postprandial CPP concentrations with CKD-MBD variables before dietary phosphate restriction

Univariable and multivariable linear regression analyses were performed to determine the associations of plasma concentrations of CPP (T-CPP, L-CPP, and H-CPP) with age, plasma concentrations of creatinine, urea, SDMA, phosphate, tCa, CaPP, tMg, total protein, albumin, ln[FGF-23], ln[PTH], PCV, and whole blood iCa concentration. The samples used for this part of the study were collected before the transition of the cats onto clinical renal diets (at the time of diagnosis of CKD).

## Results

A total of 52 euthyroid cats with naturally occurring CKD (International Renal Interest Society [IRIS] stage 2, *n* = 40; IRIS stage 3, *n* = 12) were enrolled. Domestic shorthair was the most common breed (*n* = 37), followed by domestic longhair (*n* = 9) and 1 of each of the following breeds: Burmese, Devon Rex, Norwegian Forest, Siamese, Tiffany, and Tonkinese. The median age was 15.3 (range, 7.2-20.2) years. Descriptive statistics for signalment and biochemical variables, including CPP, for the enrolled cats are presented in Table 1. Of the 52 cats, 27 (52%) were female and 25 (48%) were male. Eight (15%) cats had concurrent systemic hypertension and were treated with amlodipine. No difference in plasma CPP concentrations was found between sex (T-CPP, *P* = .24; L-CPP, *P* = .52; and H-CPP, *P* = .42) and hypertensive status (T-CPP, *P* = .16; L-CPP, *P* = .26; and H-CPP, *P* = .25) of the cats.

**Table 1:** Descriptive statistics for all enrolled cats (*n* = 52) with preprandial heparinized plasma sample available for the measurement of calciprotein particles (CPP).

**Variables (reference interval)**	**Median [25th, 75th percentile] (unless otherwise indicated)**	***n***
**Age (years)**	15.3 [12.8, 16.9]	52
**BCS (“1-3,” “4-6,” “7-9,” *n* [%])**	17 [33], 33 [63], 1 [2]	51
**MCS (“0,” “1,” “2,” “3,” *n* [%])**	2 [4], 20 [40], 20 [40], 8 [16]	50
**Weight (kg)**	3.8 [3.2, 4.6]	51
**Sex (female neutered, *n* [%])**	27 [52]	52
**Albumin (2.5-4.5 g/dL)**	3 [2.8, 3.2]	51
**ALP (≤60 U/L)**	24 [20, 33]	51
**ALT (5-60 U/L)**	50 [39, 61]	51
**Chloride (100-124 mEq/L)**	116 [115, 119]	31
**T-CPP (AU)**	13 417 [6247, 60 822]	52
**L-CPP (AU)**	22 457 [6024, 63 751]	52
**H-CPP^a^ (AU)**	0 [0, 1556]	52
**Creatinine (0.23-2 mg/dL)**	2.44 [2.18, 2.81]	51
**FGF-23 (56-700 pg/mL)**	423 [255, 780]	47
**Hypertension (controlled) (*n* [%])**	8 [15]	52
**Ionized calcium (4.76-5.48 mg/dL)**	5.28 [5.16, 5.48]	47
**PCV (30%-45%)**	34 [30, 36]	52
**Venous pH (7.21-7.44)**	7.37 [7.34, 7.39]	46
**Phosphate (2.79-6.81 mg/dL)**	3.46 [3.05, 3.92]	51
**Potassium (3.5-5.5 mEq/L)**	3.93 [3.63, 4.16]	31
**PTH^b^ (2.6-17.6 pg/mL)**	11.2 [6.7, 19.7]	36
**SBP (<160 mmHg)**	134 [125, 144]	52
**SDMA (1-14 μg/dL)**	19 [17, 21]	45
**Sodium (145-157 mEq/L)**	153 [151, 155]	31
**T_50_ (min)**	186 [162, 216]	38
**Total calcium (8.2-11.8 mg/dL)**	10.2 [9.9, 10.7]	51
**Total magnesium (1.73-2.57 mg/dL)**	2.09 [1.87, 2.21]	43
**Total protein (6.0-8.0 g/dL)**	7.8 [7.3, 8.3]	51
**Urea (7.0-27.7 mg/dL)**	46.8 [36.8, 51.7]	31
**USG (≥1.035)**	1.017 [1.015, 1.022]	24

^a^Thirty of the 52 cats had nondetectable H-CPP.

^b^Combined PTH results were obtained from immunoradiometric assay (*n* = 21) and 2-site immunoenzymatic assay (*n* = 15).

Abbreviations: *n* = number of cats; BCS = body condition score; MCS = muscle condition score; ALP = alkaline phosphatase; ALT = alanine aminotransferase; FGF-23 = fibroblast growth factor-23; H-CPP = high-density calciprotein particle; L-CPP = low-density calciprotein particle; PCV = packed cell volume; PTH = parathyroid hormone; SBP = systolic blood pressure; SDMA = symmetric dimethylarginine; T_50_ = transition time from primary to secondary calciprotein particles; T-CPP = total calciprotein particle; USG = urine specific gravity.

### Correlations of preprandial CPP concentrations and T_50_ with CKD-MBD variables after dietary phosphate restriction

Results from the univariable linear regression analyses on preprandial CPP concentrations are presented in Table S1. Moderate negative correlations were found among T-CPP, L-CPP, and detectable H-CPP with T_50_.

Plasma concentrations of ln[FGF-23], ln[PTH], tCa and CaPP, and blood iCa concentration were associated with CPP formation and transformation process in cats with CKD that had been stabilized on a standardized PRD (Tables 2 and 3; Table S1). In the final multivariable linear regression model, ln[FGF-23] and ln[PTH] were independently associated with preprandial T-CPP concentration (adjusted *R*^2^ = 0.17; Table 2, A). For preprandial L-CPP concentration, although ln[FGF-23] was included in the multivariable models, only ln[PTH] remained significant after backward elimination (adjusted *R*^2^ = 0.12; Table 2, B). Higher ln[FGF-23] was associated with increased odds (OR = 2.13 [1.02-5.02]) of having a detectable H-CPP concentration, with 10% increase in FGF-23 corresponding to 8% higher odds of detectable H-CPP (Table 3, A ).

**Table 2:** Univariable and multivariable linear regression models identifying predictors for preprandial concentrations of (A) total calciprotein particles (T-CPP), and (B) low-density calciprotein particles (L-CPP) in cats with chronic kidney disease (CKD) that are stabilized on a standardized phosphate-restricted diet.

**A**						
**Explanatory variables**	**Univariable analysis**			**Multivariable analysis**		
	**sβ (95% CI)**	***n***	***P*-value**	**sβ (95% CI)**	***n***	***P*-value**
**Ln[FGF-23]**	0.37 (0.09-0.66)	47	**.012**	0.35 (0.02-0.68)	35	**.04**
**Ionized calcium**	0.3 (0.003-0.59)	47	**.048**			
**Ln[PTH]**	−0.35 (−0.69-0.01)	36	**.047**	−0.34 (−0.67-−0.01)	35	**.042**
**Total calcium^a^**	0.29 (0.02-0.57)	51	**.036**			
**B**						
**Explanatory variables**	**Univariable analysis**			**Multivariable analysis**		
	**sβ (95% CI)**	***n***	***P*-value**	**sβ (95% CI)**	***n***	***P*-value**
**Ln[FGF-23]**	0.33 (0.04-0.62)	47	**.027**	0.34 (−0.01–0.7)	32	.056
**Ln[PTH]**	−0.42 (−0.78-0.06)	36	**.024**	−0.41 (−0.76-−0.06)	**32**	**.022**

^a^Variable is not included in the multivariable model due to collinearity issue. Significant correlations and standardized regressions (*P* ≤ .05) are highlighted in bold.

Abbreviations: *n* = number of cats; ln[FGF-23] = log-transformed fibroblast growth factor-23; ln[PTH] = log-transformed parathyroid hormone; sβ = standardized regression coefficient; 95% CI = 95% confidence interval for regression coefficient.

**Table 3:** Two-part model of predictors for high-density calciprotein particles (H-CPP) in cats with chronic kidney disease (*n* = 52). (A) Binomial logistic regression identifying predictors associated with the likelihood of detectable H-CPP compared with non-detectable H-CPP. (B) Univariable linear regression identifying predictors associated with the preprandial concentrations of H-CPP, performed only on the subset of cats with detectable concentrations (*n* = 22).

**A**			
**Explanatory variables**	**Univariable analysis**		
	**OR (95% CI)**	***n***	***P*-value**
**Ln[FGF-23]**	2.13 (1.02-5.02)	47	**.045**
**B**			
**Explanatory variables**	**Univariable analysis**		
	**sβ (95% CI)**	***n***	***P*-value**
**CaPP**	0.53 (0.13-0.92)	22	**.011**
**Ionized calcium**	0.39 (−0.08-0.86)	20	.098
**Total calcium^a^**	0.47 (0.06-0.88)	22	**.027**

^a^Variables are not included in the multivariable model due to collinearity issue. Significant associations (*P* ≤ .05) are highlighted in bold.

Abbreviations: *n* = number of cats; CaPP = calcium phosphate product; ln[FGF-23] = log-transformed fibroblast growth factor-23; OR = odds ratio; sβ = standardized regression coefficient; 95% CI = 95% confidence interval for regression coefficient.

Among the 22 cats with detectable H-CPP, tCa, iCa, and CaPP were individually associated with H-CPP concentration (Table 3, B). Both iCa and CaPP were entered into a multivariable model, only CaPP remained significantly associated with H-CPP concentration (adjusted *R*^2^ = 0.24). In addition, plasma ln[FGF-23], creatinine, albumin, and all forms of CPP were significantly associated with T_50_ in univariable analysis (Table 4). Since CPP and T_50_ are intrinsically and strongly related, CPP were not included in the multivariable model. After backward elimination, only ln[FGF-23] remained independently associated with T_50_ (adjusted *R*^2^ = 0.17; Table 4).

**Table 4:** Univariable and multivariable standardized linear regression models identifying predictors for the transition time (T_50_) from amorphous to crystalline calciprotein particles (CPP) in cats with chronic kidney disease (CKD) that are stabilized on a standardized phosphate-restricted diet.

**Explanatory variables**	**Univariable analysis**			**Multivariable analysis**		
	**sβ (95% CI)**	***n***	***P*-value**	**sβ (95% CI)**	***n***	***P*-value**
**T-CPP^a^**	−0.52 (−0.81–−0.24)	38	**<.001**			
**L-CPP^a^**	−0.41 (−0.71–−0.11)	38	**.008**			
**H-CPP^a^**			**.001**			
**Detectable vs nondetectable^b^**	−1 (−1.67–−0.32)	38	**.005**			
**Concentration of H-CPP**	−0.67 (−1.02–−0.33)	10	**.002**			
**Albumin**	0.33 (0.02–0.64)	38	**.038**			
**Creatinine**	−0.33 (−0.64–−0.03)	38	**.034**	−0.3 (−0.61–−0.01)	37	.055
**Ln[FGF-23]**	−0.48 (−0.82–−0.14)	37	**.006**	−0.37 (−0.72–−0.03)	37	**.035**

Significant correlations and standardized regressions (*P* ≤ .05) are highlighted in bold.

^a^Variables are not included in the multivariable model due to collinearity issue.

^b^Binomial logistic regression model was applied.

Abbreviations: *n* = number of cats; H-CPP = high-density calciprotein particles; L-CPP = low-density calciprotein particles; ln[FGF-23] = log-transformed fibroblast growth factor-23; sβ = standardized regression coefficient; T_50_ = transition time from amorphous to crystalline calciprotein particles; T-CPP = total calciprotein particles; 95% CI = 95% confidence interval for regression coefficient.

Another standardized linear regression analysis was performed to identify the variables associated with preprandial FGF-23 concentration in cats with CKD stabilized on a PRD. Plasma concentrations of CPP (T-CPP, L-CPP, and H-CPP), tCa and tMg, and blood iCa were significantly associated with plasma ln[FGF-23] (Table 5). In the final multivariable models, plasma creatinine, tMg and either T-CPP or L-CPP remained significant variables associated with ln[FGF-23], independent of iCa and phosphate concentrations (Table 5).

**Table 5:** Univariable and multivariable standardized linear regression models identifying predictors for log-transformed fibroblast growth factor-23 (ln[FGF-23]) in cats with chronic kidney disease (CKD) that are stabilized on a standardized phosphate-restricted diet.

**Explanatory variables**	**Univariable analysis**			**Multivariable analysis**								
				**Model 1** ^a^			**Model 2** ^b^			**Model 3** ^c^		
	**sβ (95% CI)**	***n***	***P*-value**	**sβ (95% CI)**	***n***	***P*-value**	**sβ (95% CI)**	***n***	***P*-value**	**sβ (95% CI)**	***n***	***P*-value**
**T-CPP**	0.35 (0.08–0.63)	47	**.012**	0.26 (0–0.51)	39	**.046**						
**L-CPP**	0.31 (0.04–0.59)	47	**.027**				0.26 (0.01–0.51)	39	**.045**			
**H-CPP**												
**Detectable vs nondetectable^d^**	0.6 (0–1.19)	47	**.049**									
**Concentration of H-CPP**	0.25(−0.23–0.72)	17	.285									
**CaPP^e^**	0.26 (−0.03–0.55)	46	.081									
**Creatinine**	0.26 (−0.03–0.56)	46	.079	0.33 (0.07–0.6)	39	**.016**	0.32 (0.05–0.59)	39	**.02**	0.4 (0.13–0.67)	39	**.004**
**Ionized calcium**	0.33 (0.03–0.63)	42	**.03**									
**Total calcium^e^**	0.43 (0.17–0.7)	46	**.002**									
**Total magnesium**	−0.34 (−0.63–0.06)	39	**.018**	−0.43 (−0.68–−0.17)	39	**.002**	−0.4 (−0.66–−0.14)	39	**.004**	−0.46 (−0.73–−0.19)	39	**<.001**

Significant correlations and standardized regressions (*P* ≤ .05) are highlighted in bold.

^a^T-CPP is the only form of CPP entered in the multivariable model (model 1).

^b^L-CPP is the only form of CPP entered in the multivariable model (model 2).

^c^H-CPP is the only form of CPP (as binomial) entered in the multivariable model (model 3).

^d^Binomial logistic regression model was applied.

^e^Variables are not included in the multivariable model due to collinearity issue.

Abbreviations: *n* = number of cats; CaPP = calcium phosphate product; H-CPP = high-density calciprotein particles; L-CPP = low-density calciprotein particles; sβ = standardized regression coefficient; T-CPP = total calciprotein particles; 95% CI = 95% confidence interval for regression coefficient.

### Comparison of preprandial CPP concentrations between cats with different trajectories of iCa after dietary phosphate restriction

**Figure 2:**
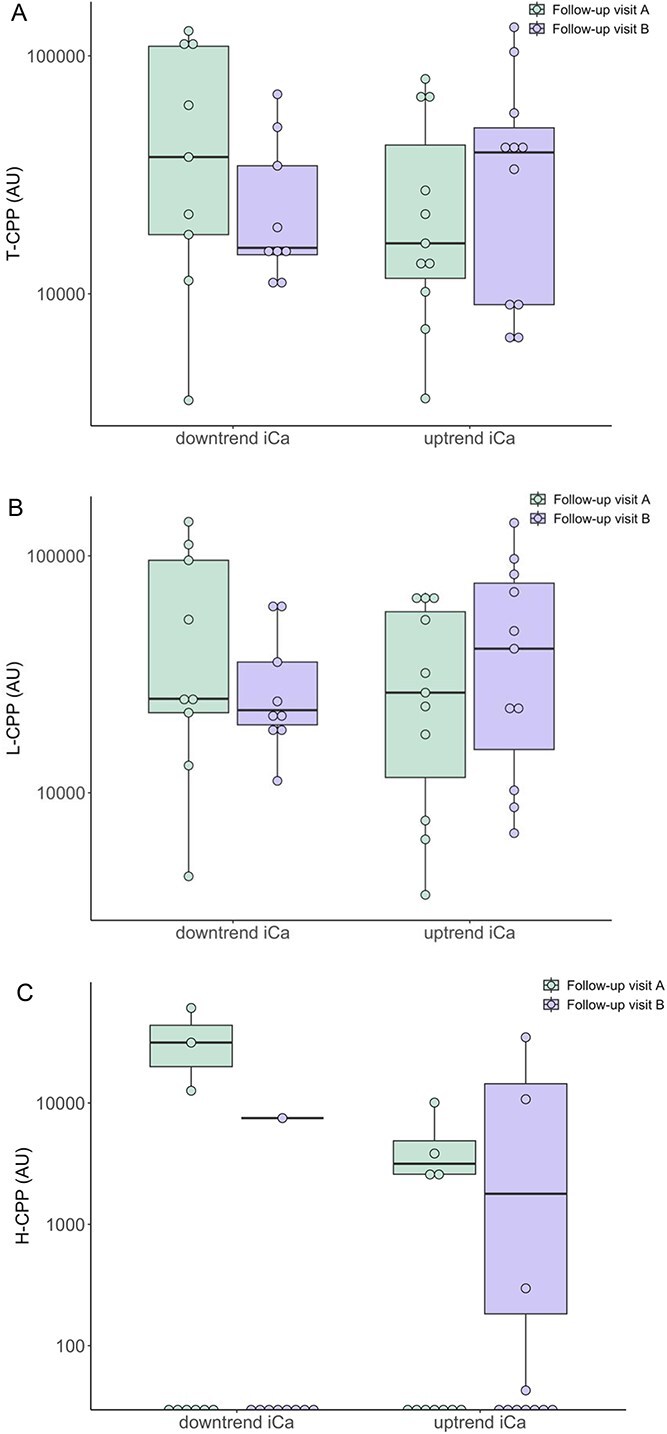
Boxplots illustrating the preprandial concentrations of (A) T-CPP, (B) L-CPP, and (C) H-CPP between groups of cats according to the trend of blood iCa concentration once they had been stabilized on a phosphate-restricted diet (“downtrend iCa” [*n* = 9] vs “uptrend iCa” [*n* = 11]) at first follow-up visit (termed follow-up visit A) and second follow-up visit (termed follow-up visit B) during the study period. Abbreviations: H-CPP = high-density calciprotein particles; iCa = ionized calcium; L-CPP = low-density calciprotein particles; T-CPP = total calciprotein particles.

Twenty-three cats with CKD had 2 follow-up visits after being stabilized on a PRD for a minimum of 4 weeks (termed “follow-up visit A” and “follow-up visit B”). Two cats did not have an iCa measurement at follow-up visit A and 1 cat did not have iCa measurement at follow-up visit B. Therefore, a total of 20 cats were included in paired analyses. The median duration between the 2 follow-up visits was 49 (IQR: 42, 65) days. Cats were grouped based on the change in blood iCa concentration between the 2 visits: cats with higher iCa at follow-up visit B compared to visit A (uptrend iCa, *n* = 11), and those with lower iCa at follow-up visit B compared to visit A (downtrend iCa, *n* = 9). There was no difference in plasma concentrations of all forms of CPP at both follow-up visits between cats with different trajectories of iCa. No significant difference in paired preprandial plasma concentrations of all forms of CPP was detected in cats with CKD with either uptrend or downtrend iCa between follow-up visits once being stabilized on a PRD (Figure 2). However, the change in T-CPP (ΔT-CPP) was significantly greater in cats with uptrend iCa compared to those with downtrend iCa between follow-up visits (14 105 ± 36 299 AU vs −29 495 ± 49 664 AU; *P* = .036; Figure S2). Similar findings on ΔL-CPP (*P* = .079) and ΔH-CPP (*P* = .061) between the 2 iCa groups were observed but statistically significance was not reached.

### Comparisons of preprandial and postprandial CPP concentrations in cats with CKD before dietary phosphate restriction

**Figure 3:**
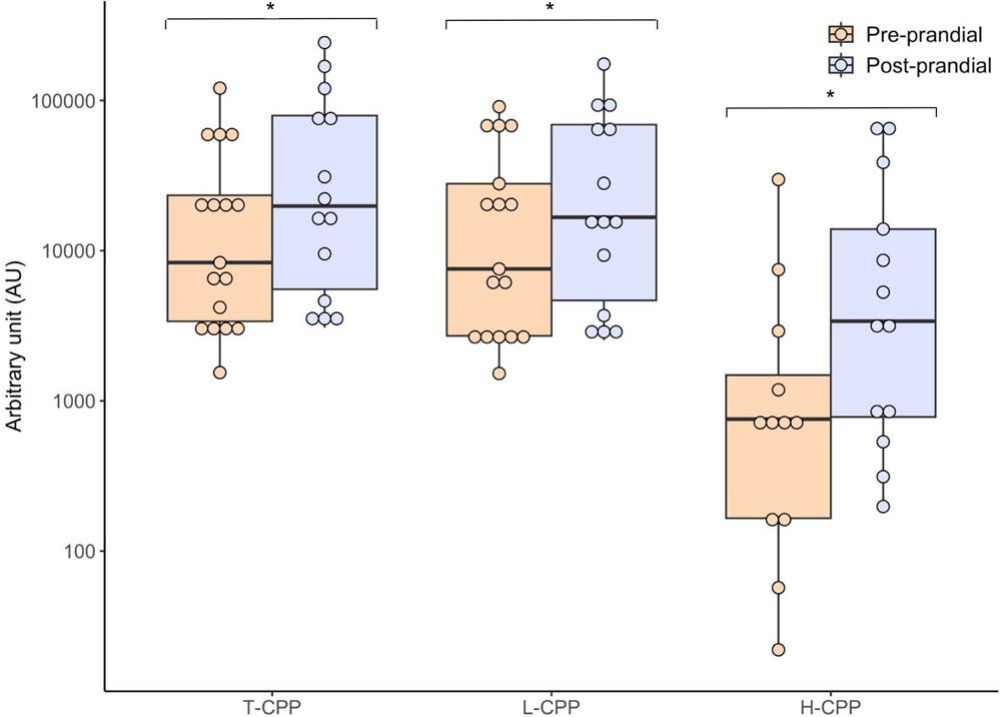
Boxplot illustrating preprandial (*n* = 17) and postprandial (*n* = 14) concentrations of T-CPP, L-CPP, and H-CPP in cats with CKD before dietary phosphate restriction. Abbreviations: CKD = chronic kidney disease; H-CPP = high-density calciprotein particles; L-CPP = low-density calciprotein particles; T-CPP = total calciprotein particles.

Of the total 52 cats with CKD, 17 cats had preprandial plasma samples available and 14 cats had plasma samples obtained postprandially before the initiation of a PRD. Seven of these were paired samples, with a median of 28 (range, 28-42) days between visits. Postprandial concentrations of all forms of CPP were significantly higher than preprandial concentrations (T-CPP, *P* = .02; L-CPP, *P* = .017; and H-CPP, *P* = .006; Figure 3). Five (out of 17) preprandial samples had nondetectable H-CPP, whereas one (out of 14) postprandial sample had nondetectable H-CPP. No difference was found in the CKD-MBD variables between preprandial and postprandial samples (Table S2).

### Correlations of postprandial CPP concentrations with CKD-MBD variables before dietary phosphate restriction

Results from the standardized univariable linear regression analyses on postprandial CPP concentrations are presented in Table S3. Strong positive correlations between all forms of CPP with plasma concentrations of ln[FGF-23]. phosphate, CaPP, and creatinine were detected.

In cats with CKD before dietary phosphate restriction, plasma phosphate concentration remained the only independent variable associated with postprandial concentrations of T-CPP (adjusted *R*^2^ = 0.48; Table 6, A) and L-CPP (adjusted *R*^2^ = 0.52; Table 6, B). For postprandial H-CPP concentration, plasma creatinine remained the only independent variable associated with H-CPP after backward elimination (adjusted *R*^2^ = 0.29; Table 6, C) .

**Table 6:** Standardized univariable linear regression models identifying variables associated with postprandial concentrations of (A) total calciprotein particles (T-CPP), (B) low-density calciprotein particles (L-CPP), and (C) high-density calciprotein particles (H-CPP) in cats with chronic kidney disease (CKD) before dietary phosphate restriction.

**A**			
**Explanatory variables**	**Univariable analysis**		
	**sβ (95% CI)**	***n***	***P*-value**
**Albumin**	−0.49 (−1.04-0.06)	14	.075
**CaPP^a^**	0.74 (0.32-1.16)	14	**.002**
**Creatinine**	0.67 (0.2-1.14)	14	**.009**
**Ln[FGF-23]**	0.71 (0.09-1.33)	10	**.029**
**Phosphate**	0.72 (0.29-1.16)	14	**.003**
**SDMA^a^**	0.59 (−0.08-1.25)	11	.076
**Urea^a^**	0.65 (0-1.29)	11	**.049**
**B**			
**Explanatory variables**	**Univariable analysis**		
	**sβ (95% CI)**	***n***	***P*-value**
**Albumin**	−0.48 (−1.03-0.07)	14	.082
**CaPP^a^**	0.77 (0.38-1.17)	14	**.001**
**Creatinine**	0.69 (0.24-1.15)	14	**.006**
**Ln[FGF-23]**	0.74 (0.13-1.36)	10	**.024**
**Phosphate**	0.75 (0.33-1.17)	14	**.002**
**SDMA^a^**	0.63 (0-1.26)	11	.051
**Urea^a^**	0.68 (0.05-1.31)	11	**.038**
**C**			
**Explanatory variables**	**Univariable analysis**		
	**sβ (95% CI)**	***n***	***P*-value**
**Albumin**	−0.49 (−1.04-0.05)	14	.072
**CaPP^a^**	0.64 (0.16-1.12)	14	**.013**
**Creatinine**	0.59 (0.08-1.1)	14	**.027**
**Ln[FGF-23]**	0.6 (0-1.2)	10	**.049**
**Phosphate**	0.63 (0.15-1.12)	14	**.015**

Significant correlations and standardized regressions (*P* ≤ .05) are highlighted in bold.

^a^Variables are not included in the multivariable model due to collinearity issue.

Abbreviations: *n* = number of cats; CaPP = calcium phosphate product; ln[FGF-23] = log-transformed fibroblast growth factor-23; SDMA = symmetric dimethylarginine; sβ = standardized regression coefficient; 95% CI = 95% confidence interval for regression coefficient.

## Discussion

Calciprotein particles have attracted increasing research interest in human CKD patients in the last decade. This study explores the concentrations of circulating CPP in cats and highlights the interlinked relationship among FGF-23, PTH, calcium, and CaPP in the formation and transformation processes of CPP in cats with CKD that are fed a standardized PRD. Significant positive associations between the different forms of CPP (T-CPP and L-CPP) with FGF-23 and PTH were identified in cats with CKD stabilized on a PRD. Detection of H-CPP and the concentration of H-CPP were associated with FGF-23 and CaPP, respectively. Preprandial concentrations of T-CPP/L-CPP, creatinine and tMg remained variables significantly associated with FGF-23 in the final multivariable models. Furthermore, our preliminary data indicated a potential difference in preprandial and postprandial CPP concentrations in a small cohort of cats with azotemic CKD. Postprandial concentrations of CPP (T-CPP and L-CPP) were primarily determined by plasma phosphate concentration in cats with CKD after being fed maintenance diets. The influence of plasma phosphate on CPP concentrations diminishes during the preprandial state, in conjunction with dietary phosphate restriction. This study provides novel insights into the associations between mineral disturbances and CPP formation in cats with CKD.

Postprandial elevation in serum phosphate is well-documented in humans^35^ and cats.^36,37^ Our study showed that postprandial CPP concentrations were significantly higher compared to preprandial concentrations in cats with CKD before dietary phosphate restriction. A human study showed a significant postprandial rise in CPM, CPP-1, and CPP-2 at 2, 3, and 4 h after a meal in both healthy controls and CKD patients.^38^ This is supported by the significant increase in serum CPP and phosphate concentrations 2 h after dietary phosphate loading in mice.^15^ The postprandial increase in circulating CPP observed in cats could potentially be a physiological response secondary to the presumed acute rise of phosphate after the ingestion of food.^37^ This phenomenon is suggested to play a pivotal role in the signaling pathway between the intestine and bone on phosphate homeostasis by regulating the expression of FGF-23 in osteoblasts. In an in vivo imaging study, intravenous injection of fluorescently labeled CPP found it to be deposited on the inner bone surface where osteoblasts reside, indicating the ability of CPP to extravasate and gain direct access to osteoblasts.^15^ An in vivo study demonstrates the presence of radioactive-labeled CPP-2 in bones after intravenous injection in both control and CKD rats using positron emission tomography.^39^ It is, therefore, proposed that CPP might act as intestinal phosphate sensor indirectly by transporting calcium and phosphate absorbed from the gastrointestinal tract to the bone. Increase in plasma CPP precedes the increase in FGF-23 after dietary phosphate loading in mice, further supporting the involvement of CPP on the regulation of FGF-23 production and secretion.^15^

Positive correlations between plasma phosphate concentration and the concentrations of CPP were observed in postprandial samples in cats with CKD. This is consistent with an earlier study in which circulating T-CPP concentration was associated with hyperphosphatemia in adenine-induced rat models with renal failure.^40^ In addition, plasma phosphate remained an independent variable associated with postprandial T-CPP and L-CPP concentrations in cats with CKD before dietary phosphate restriction, further reinforcing the hypothesis on the acute effect of dietary phosphorus load on spontaneous CPP formation postprandially. Consistent with our findings, serum phosphate concentration was the major variable associated with CPP concentrations in human CKD patients.^13^

There was diminished association between plasma phosphate and CPP concentrations in the preprandial state. In cats with CKD that have been stabilized on a PRD for a minimum of 4 weeks, preprandial concentrations of T-CPP and L-CPP were predominantly determined by plasma FGF-23 and PTH concentrations, and preprandial H-CPP were influenced by plasma FGF-23 for their detection, and by CaPP concentrations for their magnitude. A significant univariable association between FGF-23 and iCa was also observed, signifying an intertwined relationship among preprandial concentrations of FGF-23, PTH, calcium, phosphate, and CPP in cats with CKD stabilized on a PRD. Although FGF-23 was significantly associated with tCa and iCa (Table 5), these associations were no longer significant when CPP was included in the multivariable model, suggesting that CPP exerted a stronger influence on FGF-23 concentrations in cats stabilized on a PRD. The absence of a significant univariable relationship between FGF-23 and phosphate was unexpected, as phosphate is a regulator of FGF-23. This finding is likely attributed to the relatively normal phosphate concentrations (3.46 [3.05, 3.92] mg/dL) in our study cohort, where only 4 cats had plasma phosphate over 4.64 mg/dL (the target concentration for cats with IRIS stage 2 CKD), thereby limiting the ability to detect such an association. Studies examining factors associated with FGF-23 in cats primarily focus on cats at CKD diagnosis, before the introduction of a PRD, and identified plasma phosphate as an independent predictor.^31^ Since CPP might affect FGF-23 secretion in osteoblasts,^15^ it is plausible that factors contributing to CPP formation could be associated with plasma FGF-23 concentrations.

Increasing evidence reveals the association between dietary phosphate restriction and development of hypercalcemia in some cats with CKD.^41,42^ One possible explanation is that the higher dietary Ca:P ratio in PRD might enhance intestinal absorption of calcium.^41,43^ However, the possibility of enhanced calcium mobilization from bones (eg, increased bone resorption or decreased bone formation or both) in these hypercalcemic cats cannot be fully excluded. In our study, cats with CKD with uptrend iCa had a greater increase in CPP concentrations, especially T-CPP, compared to those with downtrend iCa after being stabilized on a PRD. This finding provides additional evidence that calcium disturbance could enhance CPP formation in cats with CKD. Crystalline CPP-2 induce inflammatory responses in human proximal tubule cells, leading to renal tubular damage, interstitial fibrosis, and loss of functioning nephrons.^44^ In addition, interstitial inflammation and fibrosis was found to be alleviated by the administration of bisphosphonate, which also inhibited CPP maturation.^44^ Since plasma FGF-23 and CaPP concentrations were the major variables associated with preprandial H-CPP concentrations in cats with CKD fed a PRD, it is speculated that a calcium-lowering therapy, in conjunction with dietary phosphate restriction, could reduce circulating CPP concentrations and potentially decelerate the decline in renal function in cats with CKD. In nephrectomized miniature pigs, removal of circulating CPP-2 using an alendronate column during hemodialysis improves survival and reduces the risks of VC and inflammation, despite the lack of differences in serum phosphate, calcium, CaPP, and FGF-23 observed between the treatment and control groups.^45^ Nevertheless, the clinical importance of CPP and the potential benefits of reducing CPP-2 transformation in cats with CKD require further investigation.

The transformation time (T_50_) from CPP-1 to CPP-2 reflects the propensity of calcification. Fibroblast growth factor-23 was a major variable associated with T_50_ in cats with CKD stabilized on a PRD, further reinforcing the relationship between FGF-23 and CPP. This finding is supported by a pilot study involving a total of 72 IRIS stage 2-4 cats with CKD which shows a negative relationship between serum T_50_ and plasma FGF-23 concentration.^46,47^ Similar findings were reported in human patients with stages 1-5 CKD.^48^

Our study has several limitations, mostly pertaining to its retrospective nature. The diminished significance of plasma phosphate on CPP concentrations could be attributed to the higher iCa observed in cats with CKD after the initiation of dietary phosphate restriction. However, this effect could also be attributed, at least partly, to the preprandial state. Although our study identified a significant difference between preprandial and postprandial CPP concentrations, the paired analysis showed no significant differences for any form of CPP. Only 7 cats had paired samples available for direct comparison of CPP (Figure S3), suggesting that this comparison was likely underpowered. The proposed postprandial effect on the different forms of CPP warrants further investigation through a well-controlled study where precisely timed multiple blood samples are collected after known intake of diets of different composition in a Latin-square design. As 31% of cats in our study did not have PTH measurements and 2 different assays were used, the relationship between CPP and PTH remains uncertain and warrants further investigation. In addition, CPP measurement is not universally standardized, even in human medicine. This makes it challenging not only to compare CPP values across different species but also to draw comparisons between studies employing different detection techniques. The stability of CPP in cats has not been investigated. Our plasma samples were initially stored at –20°C (up to 72 days in duration) before being transferred to –80°C before CPP measurement. The median total sample storage duration was 1572 [1172, 2216] days. A human study demonstrated minor changes in CPP values when samples were stored at –20°C compared to those stored at –80°C, but the duration of storage was not specified. Therefore, the effect of short-term storage at –20°C on our feline CPP measurements remains uncertain. Nonetheless, since all samples were collected and processed in a standardized fashion, along with the random variations in storage duration at –20°C in our study, suggest that any potential measurement biases are likely to be minimal. Lastly, given the initial exploratory nature of our study, and small sample size, any statistically significant associations identified should be interpreted with caution and warrant future evaluation in larger cohorts.

Our study demonstrated that markers closely associated with CKD-MBD (FGF-23, PTH, calcium, and CaPP) are key variables associated with various forms of CPP and T_50_ in preprandial samples from cats with CKD stabilized on a standardized PRD. Furthermore, the trajectory of iCa after dietary phosphate restriction positively correlates with changes in H-CPP in cats with CKD, emphasizing the importance in maintaining a stable and preferably normal calcium status in cats with azotemic CKD. However, the clinical implications of circulatory CPP in CKD-MBD in cats remain unclear.

## Supplementary Material

aalag037_Supplemental_Files

## Off-label antimicrobial declaration

The authors declare no off-label use of antimicrobials.

## Institutional animal care and use committee or other approval declaration

This study was approved by the Ethics and Welfare Committee of the Royal Veterinary College (URN 2023 2225-3).

## Abbreviations

Ca:P: calcium-to-phosphorus ratio
CaPP: calcium-phosphate product
CKD-MBD: chronic kidney disease-mineral and bone disorder
CKD: chronic kidney disease
CPM: calciprotein monomers
CPP-1: primary calciprotein particles
CPP-2: secondary calciprotein particles
CPP: calciprotein particles
EDTA: ethylenediaminetetraacetic acid
FGF-23: fibroblast growth factor-23
H-CPP: high-density calciprotein particles
iCa: ionized calcium
IEA: immunoenzymatic assay
IRA: immunoradiometric assay
IRIS: International Renal Interest Society
L-CPP: low-density calciprotein particles
ln: natural logarithm
Ln[FGF-23]: log-transformed fibroblast growth factor-23
Ln[PTH]: log-transformed parathyroid hormone
MBD: mineral and bone disorder
PRD: phosphate-restricted diet
PTH: parathyroid hormone
SD: standard deviation
SDMA: symmetric dimethylarginine
sβ: standardized regression coefficient
T-CPP: total calciprotein particles
T_50_: transition time from primary to secondary calciprotein particles
tCa: total calcium
VC: vascular calcification
ΔCPP: change in calciprotein particles
ΔH-CPP: change in high-density calciprotein particles
ΔL-CPP: change in low-density calciprotein particles
ΔT-CPP: change in total calciprotein particles
