# Supplementary material for: Calciprotein particles in cats with naturally occurring chronic kidney disease
Source: J Vet Intern Med. 2026 Mar 10;40(2):aalag037. doi: 10.1093/jvimsj/aalag037 (PMC12974992; doi:10.1093/jvimsj/aalag037)
Supplement: aalag037_Supplemental_Files [file aalag037_supplemental_files.zip › SUPPLEMENTARY_FIGURE_1_aalag037.docx]

**SUPPLEMENTARY FIGURE 1.** Bland-Altman plot illustrating the difference between log-transformed parathyroid hormone (lnPTH) measurements obtained from an immunoradiometric assay (IRA) and a two-site immunoenzymatic assay (IEA).


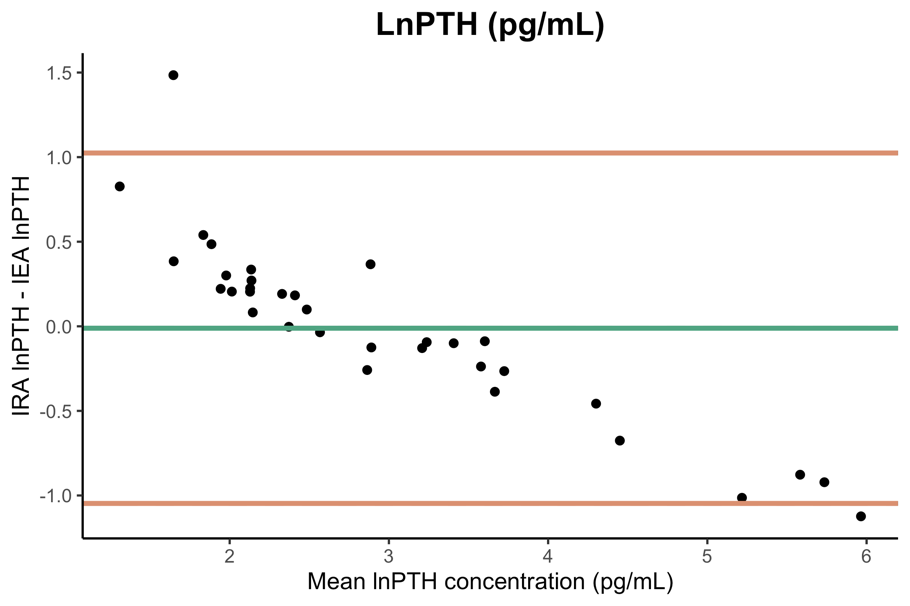


Green lines represent mean differences between methods; orange lines represent limits of agreement.

Abbreviations: IEA, immunoenzymatic assay; IRA, immunoradiometric assay; lnPTH, log-transformed parathyroid hormone.

Thirty-four feline EDTA plasma samples were selected for the comparison of parathyroid hormone (PTH) measurements obtained from an immunoradiometric assay (IRA) and a two-site immunoenzymatic assay (IEA). Both assays had previously been validated to measure plasma PTH in cats. The median plasma PTH concentrations obtained from IRA and IEA were 12.7 [9.33, 32.1] and 12.34 [7.2, 39.8] pg/mL, respectively. Wilcoxon signed-rank test identified no significant difference in PTH measurements between the two assays (*P* = 0.16). An excellent agreement in plasma PTH concentrations, especially when PTH concentrations are within the physiological levels, is illustrated in the Bland-Altman plot. However, plasma PTH concentrations obtained from the IRA appear to be consistently higher than those obtained from the IEA in cats with marked PTH excess.
