# Supplementary material for: Calciprotein particles in cats with naturally occurring chronic kidney disease
Source: J Vet Intern Med. 2026 Mar 10;40(2):aalag037. doi: 10.1093/jvimsj/aalag037 (PMC12974992; doi:10.1093/jvimsj/aalag037)
Supplement: aalag037_Supplemental_Files [file aalag037_supplemental_files.zip › SUPPLEMENTARY_FIGURE_2_aalag037.docx]

**SUPPLEMENTARY FIGURE 2.** Strip plot illustrating the change in total calciprotein particles (ΔT-CPP) between follow-up visits in cats categorized by with uptrend or downtrend in ionized calcium (iCa).


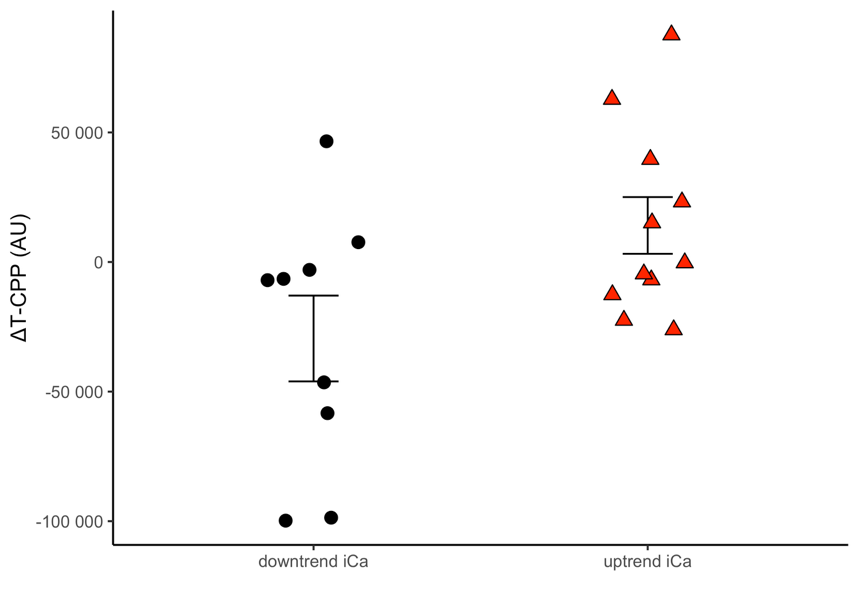


Error bars represent means ± standard error of the mean (SEM).

Abbreviations: ΔT-CPP, change in total calciprotein particles; iCa, ionized calcium.
