# Supplementary material for: Calciprotein particles in cats with naturally occurring chronic kidney disease
Source: J Vet Intern Med. 2026 Mar 10;40(2):aalag037. doi: 10.1093/jvimsj/aalag037 (PMC12974992; doi:10.1093/jvimsj/aalag037)
Supplement: aalag037_Supplemental_Files [file aalag037_supplemental_files.zip › SUPPLEMENTARY_FIGURE_3-clean_aalag037.docx]

**SUPPLEMENTARY FIGURE 3.** Paired boxplots illustrating pre-prandial and post-prandial concentrations of (A) total calciprotein particles (T-CPP), (B) low-density CPP (L-CPP), and (C) high-density CPP (H-CPP) in cats with chronic kidney disease (n = 7) before dietary phosphate restriction.


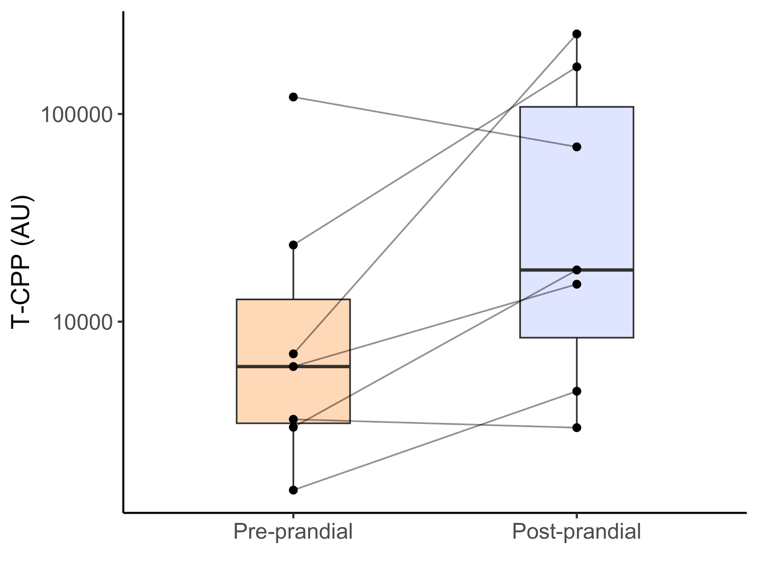


AA


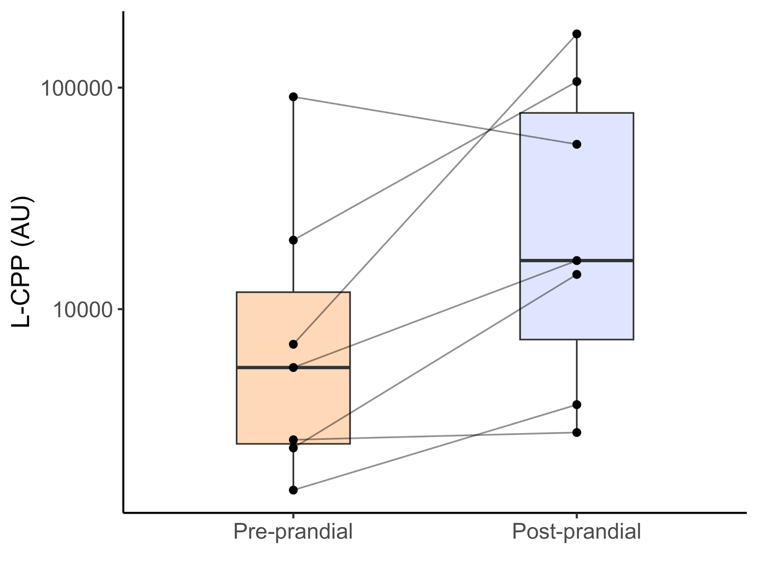


BB


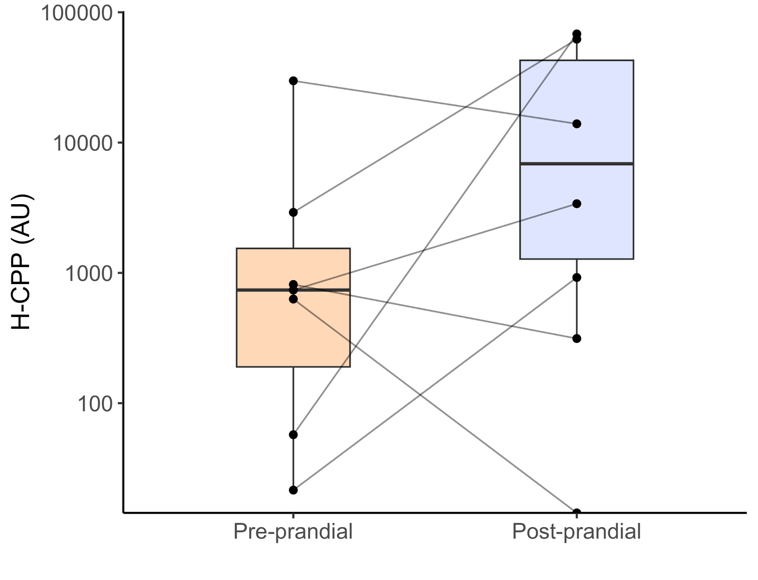


C
