# Supplementary material for: Calciprotein particles in cats with naturally occurring chronic kidney disease
Source: J Vet Intern Med. 2026 Mar 10;40(2):aalag037. doi: 10.1093/jvimsj/aalag037 (PMC12974992; doi:10.1093/jvimsj/aalag037)
Supplement: aalag037_Supplemental_Files [file aalag037_supplemental_files.zip › SUPPLEMENTARY_MATERIAL.final_aalag037.docx]

**SUPPLEMENTARY MATERIAL**

For each standardized linear regression model, the standard deviations (SD) of the significant explanatory variables (X) and outcome variables (Y) are provided below, corresponding to the models presented in Tables 2–6. Transformation of the standardized regression coefficients (sβ) back into regression coefficients (β) on the original measurement scales can be performed using the following equations:

$$\beta=s\beta\frac{SD(Y)}{SD(X)}$$

β represents the expected change in the outcome Y for per 1-unit increase in X.

When outcome variable (Y) is log-transformed (ln), for per 1-unit increase in X, we would expect to see exp(β) change in the outcome Y.

When the explanatory variable (X) is log-transformed (ln), the expected change in the response (ΔY) for proportional changes in X (for example, a 10% increase) can be calculated as:

$$\Delta Y=\beta\ln(1.1)$$

Table 2A:

The outcome variable, fasted T-CPP (n = 35), has an SD of 39,624 AU. The significant explanatory variables, ln[FGF-23] and ln[PTH], have SDs of 0.85 and 0.89, respectively.

Each 10% increase in FGF-23 corresponded to a 1,555 AU increase in T-CPP, while 10% increase in PTH corresponded to a 1,443 AU reduction in fasted T-CPP.

Table 2B:

The outcome variable, fasted L-CPP (n = 36), has an SD of 40,665 AU. The significant explanatory variable, ln[PTH], has an SD of 0.88.

Each 10% increase in PTH corresponded to a 1,850 AU reduction in fasted T-CPP.

Table 3B:

The outcome variable, fasted H-CPP (n = 22), has an SD of 15,167 AU. The significant explanatory variable, CaPP, has an SD of 8.26 mg^2^/dl^2^.

Each 1 mg^2^/dL^2^ increase in CaPP corresponded to a 973 AU increase in fasted H-CPP.

Table 4:

The outcome variable, T_50_ (n = 37), has an SD of 39.7 minutes. The significant explanatory variable, ln[FGF-23], has an SD of 0.77.

Each 10% increase in FGF-23 corresponded to a 2.4-minute decrease in T_50_.

Table 5:

The outcome variable, ln[FGF-23] (n = 39), has an SD of 0.78. The significant variables, creatinine and tMg, have SDs of 0.61 mg/dL and 0.21 mg/dL, respectively. For model 1, T-CPP has an SD of 35,353 AU. For model 2, L-CPP has an SD of 37,373 AU.

Each 1 mg/dL increase in creatinine corresponded to a 0.41–0.51 unit increase in ln[FGF-23], equivalent to approximately a 51%–67% increase in FGF-23 concentration. In contrast, each 1 mg/dL increase in tMg corresponded to a 0.15–0.17 unit decrease in ln[FGF-23], equivalent to approximately a 16%–19% increase in FGF-23 concentration. Additionally, higher CPP levels were related to higher FGF-23 concentrations, with FGF-23 increasing by 5.9% per 10,000 AU increase in T-CPP and by 5.6% per 10,000 AU increase in L-CPP.

Table 6A:

The outcome variable, post-prandial T-CPP (n = 14), has an SD of 73,747 AU. The significant variable, phosphate, has an SD of 1.81 mg/dL

Each 0.1 mg/dL increase in phosphate corresponding to increases of 2,934 AU in post-prandial T-CPP.

Table 6B:

The outcome variable, post-prandial L-CPP (n = 14), has an SD of 51,119 AU. The significant variable, phosphate, has an SD of 1.81 mg/dL.

Each 0.1 mg/dL increase in phosphate corresponding to increases of 2,118 AU in post-prandial L-CPP.

Table 6C:

The outcome variable, post-prandial H-CPP (n = 14), has an SD of 23,694 AU. The significant variable, creatinine, has an SD of 1 mg/dL.

Each 0.1 mg/dL increase in creatinine corresponding to a 1,398 AU increase in post-prandial H-CPP.

Example:

Using the model (Table 2A) with ln[FGF-23] as the explanatory variable for T-CPP as an example, the back-transformed coefficient is calculated as follows:

β = sβ × (SD[Y] / SD[X]) = 0.35 × (39624 / 0.85) = 16,316.

ΔY = β × ln[1.1] = 16,316 × 0.095 = 1,555.

Hence, a 10% increase in FGF-23 is associated with a 1,555 AU increase in fasted T-CPP.
