# Supplementary material for: Calciprotein particles in cats with naturally occurring chronic kidney disease
Source: J Vet Intern Med. 2026 Mar 10;40(2):aalag037. doi: 10.1093/jvimsj/aalag037 (PMC12974992; doi:10.1093/jvimsj/aalag037)
Supplement: aalag037_Supplemental_Files [file aalag037_supplemental_files.zip › SUPPLEMENTARY_TABLE_1.update-clean_aalag037.docx]

**SUPPLEMENTARY TABLE 1**. Standardized univariable linear regression results between pre-prandial concentrations of calciprotein particles (CPP) and clinicopathological variables in cats with chronic kidney disease (CKD) that had been stabilized on a standardized phosphate-restricted diet.

| **Explanatory variables** | **T-CPP (AU)** | | | | **L-CPP (AU)** | | | | **H-CPP (AU)^a^** | | |
| --- | --- | --- | --- | --- | --- | --- | --- | --- | --- | --- | --- |
|  | sβ (95% CI) | n | *P-* value |  | sβ (95% CI) | n | *P-* value |  | sβ (95% CI) | n | *P-*value |
| Age (years) | 0 (-0.28–0.28) | 52 | 0.998 |  | -0.09 (-0.37–0.2) | 52 | 0.541 |  | 0.02 (-0.45–0.48) | 22 | 0.939 |
| Albumin (g/dL) | 0 (-0.29–0.29) | 52 | 0.986 |  | -0.04 (-0.33–0.25) | 52 | 0.777 |  | 0.05 (-0.42–0.52) | 22 | 0.824 |
| CaPP (mg^2^/dL^2^) | 0.23 (-0.05–0.51) | 51 | 0**.**102 |  | 0.19 (-0.09–0.47) | 51 | 0.179 |  | 0.53 (0.13–0.92) | 22 | **0.011** |
| Creatinine (mg/dL) | 0.1 (-0.18–0.39) | 51 | 0.478 |  | 0.13 (-0.15–0.42) | 51 | 0.351 |  | 0.16 (-0.3–0.62) | 22 | 0.465 |
| Ln[FGF-23] | 0.37 (0.09–0.66) | **47** | **0.012** |  | 0.33 (0.04–0.62) | **47** | **0.027** |  | 0.31 (-0.28–0.9) | 17 | 0.285 |
| Ionized calcium (mg/dL) | 0.3 (0–0.59) | **47** | **0.048** |  | 0.24 (-0.05–0.54) | 47 | 0.106 |  | 0.39 (-0.08–0.86) | 20 | 0.098 |
| Ln[PTH] | -0.48 (-1.02–0.06) | 36 | 0.081 |  | -0.46 (-1.01–0.09) | 36 | 0.094 |  | -0.36 (-0.86–0.14) | 13 | 0.141 |
| PCV (%) | -0.09 (-0.37–0.19) | 52 | 0.527 |  | -0.08 (-0.36–0.2) | 52 | 0.575 |  | 0.1 (-0.37–0.56) | 22 | 0.672 |
| Phosphate (mg/dL) | 0.13 (-0.16–0.42) | 51 | 0.364 |  | 0.14 (-0.15–0.42) | 51 | 0.342 |  | 0.35 (-0.09–0.79) | 22 | 0.111 |
| SDMA (μg/dL) | -0.24 (-0.53–0.05) | 45 | 0.097 |  | -0.19 (-0.49–0.11) | 45 | 0.214 |  | -0.17 (-0.73–0.39) | 17 | 0.531 |
| Total calcium (mg/dL) | 0.29 (0.02–0.57) | **51** | **0.036** |  | 0.19 (-0.09–0.47) | 51 | 0.186 |  | 0.47 (0.06–0.88) | 22 | **0.027** |
| Total magnesium (mg/dL) | -0.03 (-0.34–0.28) | 43 | 0.832 |  | -0.1 (-0.42–0.22) | 43 | 0.518 |  | 0.21 (-0.42–0.84) | 15 | 0.486 |
| Total protein (g/dL) | 0.18 (-0.11–0.46) | 51 | 0.22 |  | 0.1 (-0.19–0.38) | 51 | 0.497 |  | 0.27 (-0.18–0.72) | 22 | 0.226 |
| T_50_ (min) | -0.53 (-0.82–(-)0.24) | **38** | **<0.001** |  | -0.43 (-0.75–(-)0.12) | **38** | **0.008** |  | -1.07 (-1.61–(-)0.52) | 10 | **0.002** |
| Urea (mg/dL) | -0.06 (-0.45–0.32) | 31 | 0.742 |  | -0.04 (-0.44–0.36) | 31 | 0.846 |  | -0.02 (-0.97–0.94) | 11 | 0.971 |

Significant standardized regressions (*P* ≤ 0.05) are highlighted in bold. T-CPP, L-CPP and detectable H-CPP concentrations are considered as outcome variables.

^a^Only the 22 of 52 cats with detectable H-CPP were included.

Abbreviations: n, number of cats; CaPP, calcium phosphate product; H-CPP, high-density calciprotein particles; L-CPP, low-density calciprotein particles; ln[FGF23], log-transformed fibroblast growth factor-23; ln[PTH], log-transformed parathyroid hormone; PCV, packed cell volume; SDMA, symmetric dimethylarginine; sβ, standardized regression coefficient; T-CPP, total calciprotein particles; T_50_, transition time from amorphous to crystalline calciprotein particles; 95% CI, 95% confidence interval for regression coefficient.
