# Supplementary material for: Calciprotein particles in cats with naturally occurring chronic kidney disease
Source: J Vet Intern Med. 2026 Mar 10;40(2):aalag037. doi: 10.1093/jvimsj/aalag037 (PMC12974992; doi:10.1093/jvimsj/aalag037)
Supplement: aalag037_Supplemental_Files [file aalag037_supplemental_files.zip › SUPPLEMENTARY_TABLE_2.update-clean_aalag037.docx]

SUPPLEMENTARY TABLE 2. Descriptive statistics comparing the pre-prandial and post-prandial concentrations of total calciprotein particles (T-CPP), low-density calciprotein particles (L-CPP), high-density calciprotein particles (H-CPP), and associated CKD-MBD parameters in cats with chronic kidney disease (CKD) before dietary phosphate restriction.

| **Variables** (reference interval) | **Pre-prandial (n = 17)** | |  | **Post-prandial (n = 14)** | |  | *P*-value |
| --- | --- | --- | --- | --- | --- | --- | --- |
|  | Median  [25^th^, 75^th^ Percentile] | n |  | Median  [25^th^, 75^th^ Percentile] | n |  |  |
| T-CPP (AU) | 8330 [3385, 23396] | 17 |  | 19928 [5851, 79594] | 14 |  | **0.02** |
| L-CPP (AU) | 7556 [2712, 27925] | 17 |  | 16696 [5110, 69646] | 14 |  | **0.017** |
| H-CPP (AU) | 169 [0, 814]^a^ | 17 |  | 3162 [595, 12602]^b^ | 14 |  | **0.006** |
| Age (year) | 15.1 [10.3, 16.6] | 17 |  | 15.9 [14.5, 16.7] | 14 |  | 0.051 |
| Albumin (2.5–4.5 g/dL) | 3.1 [2.8, 3.3] | 17 |  | 3 [2.8, 3.2] | 14 |  | 0.86 |
| CaPP (<70 mg^2^/dL^2^) | 36.8 [33.2, 53.6] | 17 |  | 40.6 [37.4, 46.6] | 14 |  | 0.27 |
| Creatinine (0.23–2 mg/dL) | 2.32 [2.12, 2.87] | 17 |  | 2.56 [2.26, 2.72] | 14 |  | 0.3 |
| PCV (30–45%) | 35 [32, 37] | 17 |  | 35 [32, 38] | 14 |  | 0.45 |
| Phosphate (2.79–6.81 mg/dL) | 3.81 [3.25, 4.37] | 17 |  | 4.06 [3.75, 4.4] | 14 |  | 0.27 |
| SDMA (1–14 μg/dL) | 17 [13, 20] | 10 |  | 18 [17, 22] | 11 |  | 0.14 |
| Total calcium (8.2–11.8 mg/dL) | 10.1 [9.6, 10.3] | 17 |  | 9.9 [9.5, 10.2] | 14 |  | 0.41 |
| Total magnesium (1.73–2.57 mg/dL) | 2.15 [1.79, 2.25] | 8 |  | 2.16 [2.03, 2.25] | 7 |  | 0.3 |
| Total protein (6.0–8.0 g/dL) | 7.7 [7.3, 8] | 17 |  | 7.9 [7.5, 8.3] | 14 |  | 0.89 |
| Urea (7.0–27.7 mg/dL) | 51 [38.9, 70.6] | 9 |  | 51.3 [44.1, 70] | 11 |  | 0.47 |

^a^Five of the 17 cats had non-detectable H-CPP in pre-prandial samples.

^b^One of the 14 cats had non-detectable H-CPP in post-prandial samples.

Significant difference between groups (*P* ≤ 0.05), as determined using Generalised estimating equations (GEE), are highlighted in bold. CPP concentrations were log-transformed before analysis.

Abbreviations: n, number of cats; H-CPP, high-density calciprotein particles; L-CPP, low-density calciprotein particles; PCV, packed cell volume; SDMA, symmetric dimethylarginine; T-CPP, total calciprotein particles.
